# Do PROMs or Sensor-Based Monitoring Detect Improvements in Patients’ Knee Function After Total-Knee Arthroplasty?—A Study Protocol for a Prospective Controlled Study

**DOI:** 10.3390/s25010118

**Published:** 2024-12-27

**Authors:** Lotanna Mba, Robert Prill, Jonathan Lettner, Nikolai Ramadanov, Robert Krause, Jan Reichmann, Roland Becker

**Affiliations:** 1 Oberlinklinik gGmbH, Orthopädische Fachklinik, Rudolf-Breitscheid-Straße 24, 14482 Potsdam, Germany; lotanna.mba@oberlinhaus.de (L.M.);; 2 Faculty of Health Sciences Brandenburg, Brandenburg Medical School Theodor Fontane, 14770 Brandenburg an der Havel, Germany; 3 Center of Orthopaedics and Traumatology, University Hospital Brandenburg/Havel, Brandenburg Medical School Theodor Fontane, 14770 Brandenburg an der Havel, Germany; 4 StatConsult GmbH, 39112 Magdeburg, Germany

**Keywords:** PROM, TKA, performance-based, knee function, rehabilitation, knee alignment, IMU

## Abstract

Determining whether preoperative performance-based knee function predicts postoperative performance-based knee function and whether patient-reported outcome measures (PROMs) completed by participants can detect these changes could significantly enhance the planning of postoperative rehabilitation for patients following total knee arthroplasty (TKA). This study aims to collect data on performance-based knee function using inertial measurement units (IMUs) worn by participants both preoperatively and postoperatively. PROMs will be completed by the patients before and after surgery to assess their ability to detect the same changes in performance-based knee function measured by the sensors. Additionally, the study will investigate the correlation between the degree of knee alignment correction and postoperative performance-based knee function in participants after TKA.

## 1. Introduction

In knee osteoarthritis (KOA), the cartilage and joint structure deteriorate, leading many patients to report difficulties in performing everyday tasks such as walking, climbing stairs, or kneeling down [1,2]. In later stages of osteoarthritis, as the cartilage is further damaged, patients experience pain even at rest, including while sitting down, or at night [3]. Furthermore, joint mobility and range of motion also suffer as most patients with end stage osteoarthritis exhibit deficits in their ability to perform knee flexion, knee extension movement, or both [4].

It is rather unknown how strong the correlation is between the expression of knee osteoarthritis (Kellgren and Lawrence grading), knee alignment deformity, and performance-based knee function (including general level of activity). Cumulative evidence suggests that combinations of structural and clinical risk factors may be able to predict radiographic knee osteoarthritis progression [5,6]. Kellgren and Lawrence grading is a widely used medical grading system to classify degrees of osteoarthritis on knee X-rays [6,7]. Grades 3 and 4 on the Kellgren and Lawrence scale are considered end-stage, at which point most patients have exhausted conservative treatment options [7,8]. Still patients with Grade 3 or 4 often benefit similarly from knee replacement surgery [9,10,11].

Patients with the symptoms and radiological signs of end-stage knee osteoarthritis are often scheduled for knee arthroplasty, mainly due to increased pain and reduced function following the breakdown of joint tissue and structure [9,12]. Depending on the location of the osteoarthritis and the severity of the symptoms and other aspects like age, either osteotomy, unicompartmental knee arthroplasty, or total knee arthroplasty may be indicated [9,13,14]. The decision-making process regarding the implantation of unicompartmental knee arthroplasty versus total knee arthroplasty involves consideration of multiple additional factors, including patient age, gender, and body mass index (BMI) [15,16].

Total knee arthroplasty is performed when a patient meets specific criteria, including symptoms and radiological signs. Also, robotic and personalized versus standard arthroplasty techniques must be considered [15,16,17,18].

Currently, there are various approaches to describing physical function in patients with knee osteoarthritis after total knee arthroplasty [19,20]. In a previous project, an investigation into how patients’ mobility was during a hospital stay was carried out with the use of new IMUs [21].

During this study, IMUs will primarily be used to track knee movement and produce data to help establish a correlation between the pre- and postoperative performance-based knee function, which will aid in determining if the former is predictive of the latter [19,20,21,22,23]. This information could serve a key role in planning postoperative rehabilitation, as well as in setting appropriate patients’ expectations during the perioperative period [14,17,24]. The expected postoperative performance-based knee function gives caregivers better insights into how to give appropriate attention in the necessary areas in the acute and postacute phase [25,26].

Patient-reported outcome measures (PROMs) are a mainstay in patient-centered medical treatment and have been employed in osteoarthritis for 35 years [27]. They allow patients to provide their own subjective report of how a treatment has affected them [28,29,30]. PROMs have a special role to play in orthopedics, especially in arthroplasty, as patients can report their ability to complete everyday tasks and compare it to their preoperative levels, as well as report on their comfort with their new prosthetic joint [31]. Furthermore, PROMs help clinicians assess the functional and psychological outcomes of surgical interventions, providing a more comprehensive evaluation of treatment success [32]. During the study, it will be of further interest whether participants will report the same degree of improvement that the IMUs identify through their subjective answers across three different PROMs. Through this study, not only will the uniformity and exchangeability of the PROMs be investigated, but their ability to reflect objective measured changes will also be examined.

Osteoarthritis of the knee can often be accompanied by one of two characteristic lower limb alignment pathologies: valgus or varus deformity [33,34]. These deformities tend to produce a more pronounced damage in either the medial or the lateral compartment of the knee [35,36]. The degree of required axis correction during total knee arthroplasty is a hotly debated topic, with mechanical, functional, and kinematic alignments taking different approaches [34,37,38]. This study will investigate the association between the degree of knee alignment correction and the postoperative performance-based knee function will be investigated. This is of interest due to the implications it could have for total knee arthroplasty planning, as surgeons can seek to provide an axis correlation most closely associated with the best performance-based knee function, rather than the previously accepted doctrine. Identifying an optimal alignment strategy may help reduce complications, improve patient satisfaction, and enhance long-term functional outcomes [39,40]. Additionally, understanding the influence of alignment correction on knee biomechanics could further refine personalized surgical approaches for osteoarthritis patients [41].

The primary hypothesis of the planned trial is that participants’ performance-based knee function two weeks pre-operation will predict their performance-based knee function six weeks post operation. The secondary hypothesis is that an improvement in performance-based knee function six weeks after total knee arthroplasty will be observed.

Additionally, it is further hypothesized that an inverse correlation between the extent of leg axis correction and knee function six weeks post operation will be observed.

## 2. Materials and Methods

### 2.1. Participants

From November 2024 till April 2025, 30 patients will be enrolled in the study. A power analysis was conducted to determine that a sample size of 30 patients would be sufficient for the study, based on the hypothesis that participants’ performance-based knee function two weeks pre-operation will predict their performance-based knee function six weeks post operation. The inclusion criteria include meeting the requirements for total knee arthroplasty in the S2k-Criteria [42] from the German Society of Orthopedics and Trauma (DGOU) summarized in Table 1, completing the preoperative examination at the ambulatory facilities of Oberlinklinik Potsdam (Germany), and informed consent to participate in the study. Exclusion criteria will include previous operations changing the knee alignment, such as high tibial osteotomies or distal femur osteotomies in the patient’s past, and comorbidities that decrease the expected knee function or activity level, such as paralysis of the lower limbs, neurological deficits in the lower limbs, and osteoarthritis in other lower limb joints.

This study has been prospectively registered in the German Clinical Trial Registry under the ID number DRKS00033537.

Ethical approval was given by the Ethics Committee of the Brandenburg Medical University (Neuruppin, Brandenburg, Germany) (ref. number: E-02-20230302). All participants will provide written consent.

### 2.2. Inertial Measurement Units

The Orthronic Smart Knee (OSK) sensor is equipped with an inertial measurement unit, incorporating a 3-axis accelerometer, gyroscope, and magnetometer, enabling the recording of knee motion at a sampling rate of 25 Hz. The OSK is equipped with a temperature sensor and an air pressure sensor, both measured at a sampling rate of 1 Hz. The sensor weighs in at 6 g and measured 37 mm × 26 mm × 8 mm (length × width × height).

The IMU can directly transmit data to a tablet via Bluetooth or store measured data in an integrated 512 MB memory bank for later access. During the development phase, the IMU’s reliability was assessed using a 10-camera motion capture system (VI-CON-MX-S, Vicon Motion System Ltd., Oxford, UK) with defined movements. This testing revealed an average deviation of 5 degrees [43].

### 2.3. IMU Application and Measurements

**Figure 1:**
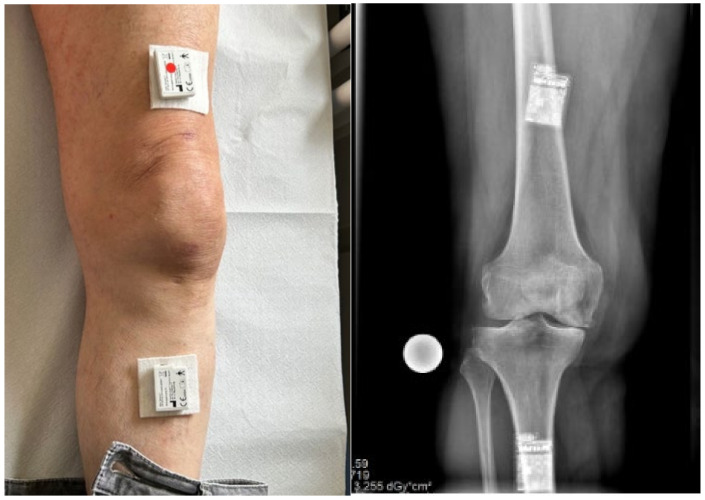
Sensors attached to a preoperative patient’ knee.

Two IMUs will be taped to the anterior distal thigh, approximately at the level of the quadriceps muscle, and on the proximal anteromedial aspect of the lower leg of each participant, respectively. As seen in Figure 1, the IMUs will be attached with the use of soft silicone adhesive non-woven fabric and cushioned from the skin with non-woven swabs. Patients will independently apply the IMUs after receiving both verbal instructions and detailed written guidelines on proper attachment. Additionally, the positioning of the sensors does not affect the measurements, as the software used to analyze data from the OSK can account for variations in sensor placement.

Each participant will wear the IMU for two sets of three-day periods, during which time they will be instructed to remove them only in the shower or bath or at night. The following factors will be measured with the IMUs.

Daily distribution of standing, horizontal, and sitting position: Each position can be identified by the orientation of the proximal or distal IMU. Standing positions will be defined as positions in which the angle between the thigh and the transverse plane is more than 45° (α > 45°). A sitting position will be defined as a position in which the angle between the thigh and the transverse plane is less than 45° while the angle between the lower leg and the transverse plane is more than 30° (α < 45° and β > 30°). A lying position will be defined as a position in which the angle between the thigh and the transverse plane is less than 12° while the angle between the lower leg and the transverse plane is less than 30° (α < 12° and β < 30°).

Movement: A significant change in the angle of the knee and movement direction is defined as a change in the knee angle by 10°. 

Steps: Steps will be counted when a standing position is detected and a change in the angel of knee of more than 20° occurs. 

Movement over angular velocity: Measuring the angle of the knee at a sampling rate of 25 Hz allows us to calculate the angular velocity using the first-time derivative below.

(1) $$Angular\;velocity=\frac{change\;of\;knee\;angle\;}{time\;interval}\times tim$$

### 2.4. PROMs

The Western Ontario and McMaster Universities Arthritis Index (WOMAC) and the Oxford Knee Score (OKS) will be used for participants to report their subjective preoperative and postoperative knee function.

#### 2.4.1. Oxford Knee Score

Comprising 12 questions, each answered using a 5-point Likert scale, this questionnaire can be self-administered by the patient. Each question has answer options from 1 (no problems) to 5 (massive problems). Thus, the total score ranges from a minimum of 12 points (reflecting the best possible outcome) to a maximum of 60 points (worst possible result and therefore highly symptomatic patient/symptomatic patient). Patients will complete the German version of the OKS questionnaire, which has been validated for the self-assessment of pain and function in patients suffering from osteoarthritis of the knee [44].

#### 2.4.2. The Western Ontario and McMaster Universities Arthritis Index

The questionnaire is composed of 33 questions, evaluating the clinical symptoms (5 questions), joint stiffness (2 questions), pain (9 questions), and activity level (17 questions). Each question has answer options from 0 (no problems) to 4 (massive problems). This results in a total maximum score of 132 points (worst possible result and therefore highly symptomatic patient/symptomatic patient) and a minimum score of 0 points (best possible result). Patients will complete the German version of the WOMAC questionnaire, which has been validated for the evaluation of symptoms and physical functional impairment in patients with knee and hip conditions [45].

### 2.5. Knee Alignment

**Figure 2:**
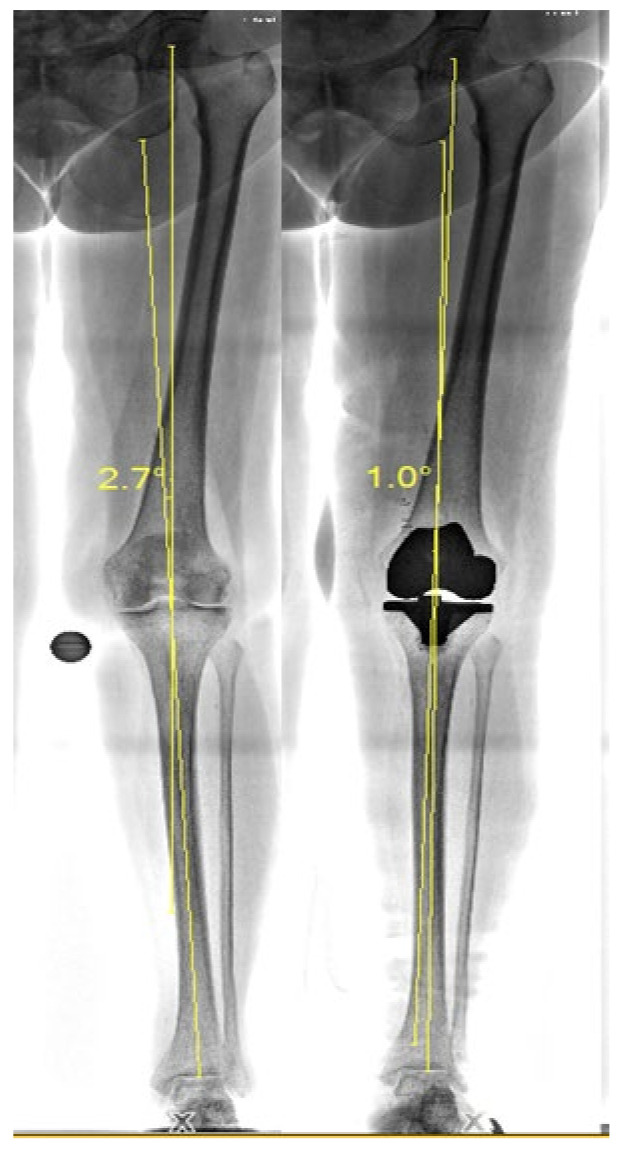
Pre- and postoperative long leg X-ray imaging with mechanical knee axis measurements.

The knee alignment for each participant will be determined pre- and postoperatively through long leg X-ray imaging. As seen in Figure 2, the mechanical axis will be measured in order to determine the degree of angulation of a participants’ knee [46].

### 2.6. Chair–Stand up Test

The Chair–Stand Up Test is used to assess the ability to stand up from a chair at normal height and sit down again, without utilizing the arms [47]. The time it takes to complete 5 repetitions of standing up from a chair is recorded and serves to give an insight into the patients’ mobility [48]. The test is well established for assessing performance during TKA rehabilitation [49,50].

## 3. Experimental Design

Over a three-day interval, occurring two weeks prior to a scheduled total knee arthroplasty, participants will be equipped with a wearable inertial movement unit (IMU). This device will facilitate the measurement of their performance-based knee function, utilizing parameters including steps taken per day, time taken to stand up, duration of active engagement, and rate of change in the knee angle. Additionally, PROMs (WOMAC and OKS) will be answered by the participants to obtain an initial measure of how they experience their own symptoms. As seen in Table 2, during the initial three-day period of wearing the inertial movement units (IMUs), the participants will also be asked to complete a Chair–Stand Up Test once a day. The data collected using the IMUs, the patient-reported outcome measures (PROMs) completed by the participants, and the results of the Chair–Stand Up Test will collectively serve to assess performance-based knee function. Preoperative leg-axis measurements will also be determined with the use of long leg X-rays. All total knee arthroplasties will be performed with kinematic alignment, utilizing an intramedullary femoral alignment and extramedullary tibial alignment. All patients will receive a cemented cruciate-retaining endoprosthesis, with a cemented posterior-stabilized (PS) tibial component, a cemented PS femoral component, and a fixed-bearing polyethylene inlay. In accordance with the guidelines established by the Institute for Quality Assurance and Transparency in Healthcare (IQTiG) for quality control, all patients will undergo physiotherapy during their hospital stay. The rehabilitation goals include sitting on the side of the bed, walking in the hospital corridors with crutches, ascending and descending stairs, and achieving knee flexion of 90 degrees. Three days post operation, knee alignment will be reassessed using long leg X-rays. At the six-week postoperative point, participants will undergo a second three-day period using IMUs to once again evaluate their performance-based knee function. During the second three-day period, participants will again perform the Chair–Stand Up Test once a day. Finally, participants will complete the two PROMs to provide a further subjective evaluation of their symptoms.

## 4. Expected Results

Throughout the study, the general expectation is an improvement in performance-based knee function in the postoperative data compared to the preoperative data.

It is expected that there will be a significant improvement in performance-based knee function (daily distribution of standing, horizontal and sitting positions, movement, steps, and movement over angular velocity) assessed with the use of the IMUs, when compared to the preoperative three-day monitoring period. Furthermore, it is hypothesized that the preoperative performance-based knee function will serve as a predictor for postoperative performance-based knee function.

It is also suspected that the postoperative improvement in performance-based knee function will also be reflected in the results of the Chair–Stand Up Test, with an anticipated decrease in the time required to complete the five repetitions.

Moreover, it is expected that the PROMs filled out by the patients pre- and postoperatively will reflect the improvement in performance-based knee function, as patients may adjust their answers to the questions on both WOMAC and OKS.

Finally, it is expected that the degree of knee alignment will have an inverse correlation with the performance-based knee function during the postoperative three-day monitoring period.

## 5. Discussion

There exist numerous long-term implications associated with closely monitoring patients through the utilization of inertial movement units (IMUs). Preoperatively, such monitoring serves the purpose of evaluating the progression of degenerative changes occurring in the knee joint. Through multiple monitoring intervals spanning several years, it becomes feasible to track patients’ performance-based knee function, thereby enhancing the ability to ascertain the optimal timing for intervention. This longitudinal approach enables healthcare providers to not only detect early signs of functional decline but also to make data-driven decisions regarding surgical or non-surgical treatment strategies, ensuring personalized care tailored to the patient’s unique trajectory of joint deterioration.

Additionally, IMU monitoring holds promise as an adjunctive tool for classifying and stratifying patients with osteoarthritis. This capability aids in grouping patients according to their functional capacities, enabling clinicians to implement targeted treatment plans. For instance, individuals exhibiting slower progression of joint degeneration may benefit from continued conservative management, while those showing rapid functional decline could be prioritized for surgical intervention. Moreover, the effectiveness of conservative therapies can be gauged by monitoring patients’ performance-based knee function both before and after initiating treatments such as oral pain medications, physical therapy regimens, or intra-articular injections. By objectively measuring changes in knee movement patterns, IMUs can offer insights into the efficacy of various interventions, enabling timely adjustments in treatment strategies to maximize patient outcomes.

The precision of IMUs in tracking intricate movements of the knee joint offers notable advantages for postoperative rehabilitation. Specifically, rehabilitation efforts can be tailored to address specific deficiencies in a patient’s knee movement. Preoperatively, individuals experiencing challenges with isolated flexion or extension can receive targeted attention in these particular areas, ensuring that the rehabilitation process is informed by a thorough understanding of the patient’s baseline function. This precision continues into the postoperative phase, where IMU monitoring allows for real-time adjustments to rehabilitation protocols, focusing on the patient’s evolving needs as they progress through recovery. Postoperative rehabilitation can be augmented through IMU tracking, enabling an individualized assessment of each patient’s rehabilitation requirements, ensuring that therapy is neither under- nor over-prescribed.

IMU data facilitate the evaluation of rehabilitation requirements for individual patients by providing objective feedback on joint kinematics, muscle activation patterns, and overall movement efficiency. Based on the collected IMU data, rehabilitation programs can offer varying intensities of therapy. Patients exhibiting sustained performance-based knee function may not necessitate intensive rehabilitation, while those demonstrating more limited performance-based knee function can benefit from more rigorous physiotherapy interventions aimed at achieving their desired knee function objectives. By dynamically adjusting the rehabilitation program in response to continuous monitoring, clinicians can promote faster recovery times while minimizing the risk of overexertion or injury.

Additionally, the integration of IMUs can facilitate the identification of patients at risk of falls, stemming from factors such as instability or other underlying conditions. IMU-generated data allow clinicians to detect subtle patterns of instability, muscle weakness, or abnormal gait mechanics that may not be visible through traditional observational methods. Preoperative IMU data can serve as an indicator for necessitating heightened patient supervision during their hospital stay and subsequent rehabilitation period. This proactive approach enables healthcare providers to implement appropriate precautions and interventions to mitigate the risk of falls and ensure patient safety throughout their care journey. In particular, patients who exhibit poor balance or decreased mobility during preoperative monitoring may benefit.

## Figures and Tables

**Table 1:** S2k-Criteria for total knee arthroplasty (TKA) from the DGOU.

Main Criteria	Details
Knee Pain	Presence of significant knee pain.
Evidence of Structural Damage	Osteoarthritis (Kellgren and Lawrence Grade 3 or 4 in weight-bearing X-rays).Osteonecrosis with resulting joint surface deformity or defect.
Failure of Conservative Therapy	Insufficient response to a tailored combination of pharmacological and non-pharmacological conservative treatments according to the osteoarthritis guideline.
Disease-Related Impairment of Quality of Life	Disease-related impairment in quality of life persisting for at least 3 months.
Subjective Disease-Related Distress	Presence of subjective distress related to the knee joint disease.
Pain-Related Functional Impairment	Pain intensity, duration, frequency, and response to conservative therapy are significant factors in therapy decision-making.
Assessment of Distress and Quality of Life	Validated patient-reported outcome instruments (both disease-specific and generic) should be used to evaluate distress and impaired quality of life.

**Table 2:** Timeline of events in the data collection.

	Event 1 (2 Weeks Preop)	Event 2 (3 Days Postop)	Event 3 (6 Weeks Postop)
PROMs (WOMAC and OKS)	X	-	X
IMUs (3 days continuously)	X	-	X
Knee alignment determination	X	X	-
Chair–Stand Up Test	X	-	X

## Abbreviations

Abbreviation	Definition
PROM	Patient-reported outcome measures
TKA	Total knee arthroplasty
IMU	Inertial measurement units
OSK	Orthronic Smart Knee
BMI	Body Mass Index
WOMAC	Western Ontario and McMaster Universities Arthritis Index
OKS	Oxford Knee Score
DGOU	Deutschen Gesellschaft für Orthopädie und Unfallchirurgie e.V./German Society of Orthopedics and Trauma
PS	posterior stabilized
IQTiG	Institut für Qualitätssicherung und Transparenz im Gesundheitswesen/Institute for Quality Assurance and Transparency in Healthcare
